# Generalized mean *p*-values for combining dependent tests: comparison of generalized central limit theorem and robust risk analysis

**DOI:** 10.12688/wellcomeopenres.15761.1

**Published:** 2020-03-31

**Authors:** Daniel J. Wilson

**Affiliations:** 1 Big Data Institute, Nuffield Department of Population Health, University of Oxford, Oxford, OX3 7LF, UK

**Keywords:** Combined tests, p-values, generalized means, generalized central limit theorem, robust risk analysis, harmonic mean p-value, dependent tests

## Abstract

The test statistics underpinning several methods for combining *p*-values are special cases of generalized mean *p*-value (GMP), including the minimum (Bonferroni procedure), harmonic mean and geometric mean. A key assumption influencing the practical performance of such methods concerns the dependence between *p*-values. Approaches that do not require specific knowledge of the dependence structure are practically convenient. Vovk and Wang derived significance thresholds for GMPs under the worst-case scenario of arbitrary dependence using results from Robust Risk Analysis (RRA).

Here I calculate significance thresholds and closed testing procedures using Generalized Central Limit Theorem (GCLT). GCLT formally assumes independence, but enjoys a degree of robustness to dependence. The GCLT thresholds are less stringent than RRA thresholds, with the disparity increasing as the exponent of the GMP ( *r*) increases. I motivate a model of *p*-value dependence based on a Wishart-Multivariate-Gamma distribution for the underlying log-likelihood ratios. In simulations under this model, the RRA thresholds produced tests that were usually less powerful than Bonferroni, while the GCLT thresholds produced tests more powerful than Bonferroni, for all *r*> − ∞. Above *r*> − 1, the GCLT thresholds suffered pronounced false positive rates. Above *r*> − 1/2, standard central limit theorem applied and the GCLT thresholds no longer possessed any useful robustness to dependence.

I consider the implications of these results in the context of various interpretations of GMPs, and conclude that the GCLT-based harmonic mean *p*-value procedure and Simes' (1986) test represent good compromises in power-robustness trade-off for combining dependent tests.

## 1 Introduction

Combining *p*-values is a convenient and widely used form of meta-analysis that aggregates evidence across studies or tests, e.g. 1, 2. Aggregating evidence in this way improves the sensitivity (power) of formal tests to detect subtle signals in data, making better use of resources and improving the potential for scientific discovery. There are many methods for combining tests, e.g. 3, 4. In general, combining tests using the full data is more powerful than using summary statistics. However, access to the full data may be difficult for many reasons, for example computational tractability or issues of consent in statistical genetics. For these reasons, parameter estimates and standard errors, or *Z*-statistics, are often provided instead, e.g. 5. Combining *Z*-statistics, rather than *p*-values, allows parameters to be jointly estimated across datasets, e.g. 6. Nevertheless, combining *p*-values may be preferred when: (i) parameters are dataset-specific, (ii) hypotheses are mutually exclusive, or (iii) only the *p*-values are available. Fisher’s is a widely-used method in scenario (i) that is appropriate when the datasets are independent ^7^. The harmonic mean *p*-value (HMP) is suited to scenario (ii) ^8, 9^. The Bonferroni procedure ^10^ is a universal method for combining *p*-values under arbitrary dependence. These methods are closely connected and can be thought of as occupying different strategies in trading off power against robustness to dependence.

Box 1. **Kinds of combined tests for *p*-values**

Some methods for combining *p*-values were originally formulated as constructing simultaneous confidence intervals for multiple tests, including the Bonferroni and Šidák procedures ^10, 11^. They account for multiple comparisons by widening the confidence interval of each test from 100(1 − )% to 100(1 – / *K*)% and 100(1 – ) ^1/ *K*^ %, assuming arbitrary dependence and independence respectively, where *K* is the number of tests. A *p*-value can be defined as the widest 100(1 – *p*)% confidence interval that rejects the null hypothesis. Therefore, these approaches are equivalent to increasing the stringency of the *p*-value threshold of each test from to /K and 1 – (1 – ) ^1/ *K*^ respectively. When any individual *p*-value falls below the adjusted threshold, the grand null hypothesis that none of the tests are significant can be rejected. Adjusting confidence intervals and significance thresholds for multiple testing is thus equivalent to a combined test in which the minimum *p*-value is compared against the adjusted significance threshold. For instance, Tippett’s combined test ^12^, in which the minimum *p*-value is compared to significance threshold 1 – (1 – ) ^1/ *K*^ is equivalent to Šidák correction for this reason. In this article, I do not distinguish between these formulations of combining *p*-values. All aim to limit the probability of falsely rejecting the grand null hypothesis that none of the individual tests is significant, at a pre-determined level (the weak-sense family-wise error rate ^13^). Some combined tests (e.g. 9– 11) are additionally able to reject subsets of tests when some null hypotheses are false while limiting the probability of falsely rejecting any true null hypotheses (the strong-sense family-wise error rate ^13, 14^).

The focus of this article is to consider significance thresholds for the generalized mean *p*-value (GMP) and compare their performance under different dependence assumptions. The test statistics underpinning the Bonferroni, Šidák, HMP, Fisher and other procedures are special cases of the GMP:

$$M_{r,K}(p_{1},\text{...},p_{K})={\left(\frac{p_{1}^{r}+\cdots +p_{K}^{r}}{K}\right)}^{1/r}\;(1)$$

which includes the maximum (when *r* → ∞), arithmetic mean ( *r* = 1), geometric mean ( *r* → 0), harmonic mean ( *r* = –1) and minimum ( *r* → –∞) ^15^. The exponent parameter *r* affects the characteristics of the test, so that as *r* approaches –∞, the GMP is more influenced by smaller *p*-values, and as it approaches ∞ is it more influenced by larger *p*-values. These characteristics affect the interpretation of the GMP as suitable for particular purposes, such as model averaging (HMP; *r* = –1), or combining evidence (Fisher’s method; *r* → 0). (See section 6 for more on interpretation).

In general, GMPs cannot be interpreted directly as *p*-values because they are not uniformly distributed even when the null hypothesis (that the constituent *p*-values are uniformly distributed) is true ^15^. Instead the GMP can be used as a test statistic by calculating a significance threshold Ψ _*r,K*_() for rejecting the null hypothesis, which limits the false positive rate to some pre-specified level, e.g. = 5%:

$$\text{Pr}\left(M_{r,K}(p_{1},\text{...},p_{K})\leq \Psi_{r,K}()|\overset{K}{\underset{k=1}{\cap }}p_{k}∼U(0,1)\right)\leq \;\text{.}\;(2)$$

This requires an assumption about dependence between the constituent *p*-values. For example, the *p*-values may be assumed (i) to be independent, as in Fisher’s method ^7^, (ii) to conform to a particular model of dependence, as in Brown’s method ^16^, or (iii) to possess arbitrary dependence, in which the worst case is usually considered, as in the Bonferroni procedure ^10^.

Different assumptions about dependence produce different significance thresholds, which in turn affect the power of the test to reject the null hypothesis. More conservative assumptions produce more stringent thresholds that trade off reduced statistical power against greater robustness of the false positive rate to dependence. Vovk and Wang ^15^ derived significance thresholds for GMPs under arbitrary dependence by considering the worst case scenario using robust risk analysis (RRA). For the HMP they derived a significance threshold of

$$\Psi_{\text{RRA},-1,K}()=\frac{}{\text{log}\;K}\;(3)$$

assuming large *K*. The result is precise (asymptotically as *K* → ∞) in the sense that for any value of , there is a form of dependence under which the threshold is not conservative (so the equality in Equation 2 is satisfied) ^15^.

In contrast, Wilson ^9^ derived a considerably less stringent threshold using generalized central limit theorem (GCLT):

$$\Psi_{\text{GCLT},-1,K}()=\frac{{}_{1}}{1+{}_{1}\text{log}\;K},\;(4)$$

where _1_ ≈ and *K* is assumed large. This result implies that the HMP can be directly interpreted as if it were a *p*-value when it is small, because

$$\Psi_{\text{GCLT},-1,K}()\to \;\text{as}\;\to 0.\;(5)$$

The difference in the stringency of the GCLT and RRA thresholds, which approaches log *K* for small , stems from different assumptions about dependence between the constituent *p*-values. Formally, the GCLT derivation assumes independence, but the heavy-tailed distribution of *p* ^–1^ confers robustness to dependence ^9, 17^. Specifically, a result by Davis and Resnick ^18^ implies that Equation 5 holds despite dependence subject to the condition that

$$\text{Pr}\left(p_{j}<|p_{i}<\right)\to 0\;\text{as}\;\to 0,\;i\neq j,\;i,j=1,\ldots ,K\text{.}\;(6)$$

However, Goeman, Rosenblatt and Nichols ^19^ reported simulations under a model of dependence satisfying the Davis-Resnick condition in which the GCLT threshold for = 0.05 incurred a false positive rate of 0.09. This raises several questions:

1. What forms of dependence are relevant when combining *p*-values?
2. Does the Davis-Resnick condition confer on the GCLT threshold adequate robustness to such dependence for practically relevant values of , e.g. 0.05?
3. Do GMPs with exponents *r* ≠ –1 enjoy a more favourable power-robustness trade-off?

To address these questions, I derived significance thresholds for GMPs using GCLT ( section 2). I motivated a model of *p*-value dependence based on the Wishart-Multivariate-Gamma distribution ( section 3). I simulated under this model to test the power and false positive rates of the GCLT and RRA significance thresholds ( section 4). To complete the picture, I derived procedures to control the strong-sense family-wise error rate based on the GCLT thresholds ( section 5) and considered the interpretation of combining *p*-values using GMPs ( section 6). The results indicate that the power of the GMP to combine *p*-values, under relevant dependence assumptions at = 0.05, was better than the Bonferroni procedure for GCLT thresholds, but worse than the Bonferroni procedure for RRA thresholds. However, GCLT thresholds began to suffer pronounced false positive rates for *r* > –1, and enjoyed apparently no robustness to dependence whatever for *r* > –1/2. I conclude that the GCLT-based HMP procedure ^9^ and the related Simes (1986) test ^20^ represent good compromises in power-robustness trade-off for combining dependent *p*-values. These methods are interpretable in terms of model-averaging and require no specific knowledge of the dependence structure.

## 2 GCLT significance thresholds for generalized mean *p*-values

This section uses GCLT to infer the distribution of GMPs under the grand null hypothesis and thereby construct significance thresholds, assuming the number of constituent *p*-values *K* is large. The GCLT derivation formally assumes the *p*-values are independent, but the Davis-Resnick condition extends to –∞ < *r* < 0, which implies that robustness to dependence is expected for small . The case *r* = 0 (geometric mean) cannot be directly attained by the same GCLT approach, but Fisher’s method provides the exact solution anyway.

Define the random variables $X_{i}=p_{i}^{r}$ and

$$Y=X_{1}\text{+}\cdots \text{+}X_{K}=K\;M_{r,K}{\left(p_{1}\text{,}\ldots ,p_{K}\right)}^{r}\cdot \;(7)$$

Assuming independence between *p*_1_, . . . , *p _K_*, GCLT states ^21^ that

$$\frac{Y-a_{r,K}}{b_{r,K}}$$

converges to a Stable distribution with heavy tail index *λ* > 0, where *λ* ≥ 2 corresponds to the Normal distribution. The heavy tail index is determined by the tails of the individual *X _i_*s, characterised as

$$\;\text{Pr}(X_{i}>x)\approx cx^{-\lambda },\;x\to \infty \;(8a)$$

$$\text{Pr}(X_{i}\;\text{<}\;\text{−}x)\approx d{\left|x\right|}^{-\lambda },\;x\to \infty \;(8b)$$

Assuming that *p* ~ *U* (0, 1),

$$\text{Pr}(X_{i}>x)\;=\;\text{Pr}(p_{i}^{r}>x)\;$$

$$\;=\;\left\{ \begin{array}{l} \text{Pr}(p_{i}>x^{1/r})\;\mathrm{if}\;r>0\\ \text{Pr}(p_{i}<x^{1/r})\;\mathrm{if}r<0 \end{array}\right.\;$$

$$\;=\;\left\{\begin{array}{l} 1-x^{1/r}\;\mathrm{if}r>0\mathrm{and}0\leq x\leq 1\\ \;x^{1/r}\;\mathrm{if}r<0\mathrm{and}x\geq 1\\ \;0\;\text{otherwise} \end{array}\;(9\right)$$

In other words,

$$X_{i}∼\left\{ \begin{array}{l} \;\text{Beta}(1/r,1)\;\mathrm{when}r>0\\ \text{Pareto}(1,-1/r)\;\mathrm{when}r<0 \end{array}\right.\;(10)$$

Thus *c* = 1, *d* = 0 and

$$\lambda =\left\{ \begin{array}{l} \;\infty \;\mathrm{if}\;r>0\\ -1/r\;\mathrm{if}\;r<0 \end{array}\right.\;(11)$$

Uchaikin and Zolotarev Table 2.1 ^21^ gives coefficients for the corresponding Extremal Stable distributions that occur when ( *c* – *d*)/( *c* + *d*) = 1 ( Table 1). In Table 1, *S _λ_*, _1_ is an Extremal Stable distribution in Nolan’s S1 parameterization (Equation 1 of 22) with parameters *α* = *λ*, *β* = 1, *σ* = 1, *µ* = 0. This is equivalent to Nolan’s S0 parameterization (Equation 3 of 22 but it seemed to me there was a missing factor of *i* on line 2, second term in square brackets) with parameters *α* = *λ*, *β* = 1, *σ* = 1 and, if *α* = 1, *µ* ^0^ = 0, or if *α* ≠ 1, *µ* ^0^ = *β* tan( *πλ*/2).

**Table 1.** Generalized central limit theorem results for non-negative random variables ^21^.

*r*	*λ*	*a _r,K_*	$C_{r,K}=b_{r,K}/K^{\text{max}\{\frac{1}{2},-r\}}$	$\frac{1}{K}\sum_{i=1}^{K}X_{i}$
*r* < − 1	0 < *λ* < 1	0	${\left[\frac{2}{\pi }\Gamma (-\frac{1}{r})\text{sin}(-\frac{\pi }{2r})\right]}^{r}$	Cr,KK−r−1S−1r,1
*r* = − 1	*λ* = 1	*K* log *K*	$\frac{\pi }{2}$	$\frac{\pi }{2}S_{1,1}+\text{log}\;K$
$-1<r<-\frac{1}{2}$	1 < *λ* < 2	*K* [ *X*]	${\left[\frac{2}{\pi }\Gamma (-\frac{1}{r})\text{sin}(-\frac{\pi }{2r})\right]}^{r}$	Cr,KK−r−1S−1r,1+[X]
$r=-\frac{1}{2}$	*λ* = 2	*K* [ *X*]	(log *K*) ^1/2^	$\sqrt{\frac{\text{log}\;K}{K}}S_{2,1}+[X]$
$-\frac{1}{2}<r<0$ *r* > 0	*λ* > 2	*K* [ *X*]	([ *X*]/2) ^1/2^	$\sqrt{\frac{[X]}{2K}}S_{2,1}+[X]$

The moments required by Table 1 are

$$\left[X\right]=\left\{ \begin{array}{l} \;\infty \;\mathrm{if}\;r\leq -1\\ \frac{1}{1+r}\;\mathrm{if}\;r>-1 \end{array}\right.\;(12)$$

$$\left[X\right]=\left\{ \begin{array}{l} \;\infty \;\mathrm{if}\;r\leq -\frac{1}{2}\\ \frac{r^{2}}{{\left(1+r\right)}^{2}(1+2r)}\;\mathrm{if}\;r>-\frac{1}{2} \end{array}\right.\;(13)$$

The main result of this paper is that a general significance threshold for the GMP with exponent *r* based on GCLT is therefore

ΨGCLT,r,K()={[ar,K+br,KF−1r,1−1(1−)K]1/rifr<0[ar,K+br,KF2,1−1()K]1/rifr>0(14)

where $F_{\lambda ,1}^{-1}$ is the inverse cumulative distribution function of an Extremal Stable random variable *S _λ_*, _1_ and the coefficients *a _r,K_* and *b _r,K_* are defined in Table 1. It is notable that for any $\lambda >2,\;S_{\lambda ,\text{1}}\;\overset{d}{=}\;S_{2,1},$ which is a Normal(0, 2) distribution. This occurs when *r* > –1/2 because the *p ^r^*s are no longer heavy-tailed in the sense that their variances are defined. Those results are therefore equivalent to a straightfoward application of central limit theorem. This transition in the behaviour of the GMP has implications for its robustness to dependence.

### 2.1 Small approximation

By the theory of regularly varying functions, the general significance threshold ( Equation 14) simplifies when $r<-\frac{1}{2}$ (i.e. *λ* < 2) to

$$\Psi_{\text{GCLT},r,K}()\to \frac{}{K^{1+1/r}}\;\text{as}\;\to 0\;(15)$$

because, (see Davis and Resnick ^18^ Lemma 2.1)

$$\begin{array}{l} \mathrm{Pr}\left(M_{r,K}(p_{1},\ldots ,p_{K})<\frac{}{K^{1+1/r}}\right)\;=\;\text{Pr}\left(K\;M_{r,K}{\left(p_{1},\ldots ,p_{K}\right)}^{r}>K^{-r}\;{}^{r}\right)\\ \;=\;\text{Pr}(p_{1}^{r}+\cdots +p_{K}^{r}>K^{-r}\;{}^{r})\\ \;\to \;K\;\text{Pr}(X>K^{-r}\;{}^{r})\;\text{as}\;\to 0\\ \;=\;K{\left(K^{-r}{}^{r}\right)}^{1/r}=. \end{array}\;(16)$$

subject to the Davis-Resnick condition ( Equation 6).

The small approximation shows that the HMP is the only GMP that can be directly interpreted as if it were a *p*-value, and only then when → 0. The small approximation is compared to the significance thresholds of Vovk and Wang ^15^ in Table 2.

**Table 2.** Significance thresholds for *M*_*r*, *K*_ ( *p*_1_, . . . , *p _K_*) assuming large *K* and, for the GCLT threshold, small .

*r*	*λ*	$\Psi_{\text{RRA},r,K}()$ ^15^	$\Psi_{\text{GCLT},r,K}()$ as → 0 ( Equation 15)
*r* < –1	0 < *λ* < 1	$\frac{}{\frac{r}{r+1}K^{1+1/r}}$	$\frac{}{K^{1+1/r}}$
*r* = –1	*λ* = 1	$\frac{}{\text{log}\;K}$	
$–1<r<–\frac{1}{2}$	1 < *λ* < 2	$\frac{}{{\left(r+1\right)}^{1/r}}$	$\frac{}{K^{1+1/r}}$
$r\geq –\frac{1}{2}$	*λ* ≥ 2	$\frac{}{{\left(r+1\right)}^{1/r}}$	no small approx.

Equation 16 appears to be applicable in the transitional region $-\frac{1}{2}\leq r<0\;(\text{i.e.}\;\lambda \geq 2)$ but here the tail behaviour can be alternatively characterised as Gaussian. Empirically, both approximations appear to struggle, so I caution that the small approximation is not helpful for $r>-\frac{1}{2}$.

For small , the GCLT significance threshold is less stringent than the RRA significance threshold by a factor of *r*/(1 + *r*) when *r* < –1, log *K* when *r* = –1 and ( *r* + 1) ^1/ *r*^/ *K* ^1+1/ *r*^ when –1 < *r* < –1/2. As *r* → –∞, the GCLT and RRA thresholds converge to those of the Šidák and Bonferroni procedures respectively, which are equivalent for small . Even below *r* = –2, the difference in stringency is less than two-fold when is small, suggesting that in this region, the approaches are similar. However, directly comparing the significance thresholds only allows a comparison of the extreme cases of independence and arbitrary dependence. More generally, an explicit model of *p*-value dependence is required, which is the subject of the next section.

## 3 Dependence structure of likelihood ratio tests

In motivating a dependence structure for *p*-values, I consider the *p*-values to have arisen from nested likelihood ratio tests, in which each *p*-value is a regularly varying function of the maximized likelihood ratio *R _i_* for a pair of nested models *ℳ*_0_ and *ℳ _i_*. Asymptotic theory for classical inference states (under various assumptions ^23^) that the deviance equals

$$2\;\text{log}\;R_{i}\approx {\text{S}}_{i}^{\prime }{\text{S}}_{i}\;(17)$$

$$\;={\left({\left[{\hat{\theta }}_{i}\right]}^{-1/2}({\hat{\theta }}_{i}-\theta_{0i})\right)}^{\prime }\left({\left[{\hat{\theta }}_{i}\right]}^{-1/2}({\hat{\theta }}_{i}-\theta_{0i})\right),\;(18)$$

where ${\hat{\theta }}_{i}$ is the maximum likelihood estimate of the *v _i_* parameters to be estimated under model *ℳ _i_* but not *ℳ*_0_, ***θ***_0 *i*_ are their assumed values under *ℳ*_0_, and $[{\hat{\theta }}_{i}]$ is the variance-covariance matrix of the maximum likelihood estimate. Under the usual assumptions, $[{\hat{\theta }}_{i}]$ is a function of the Fisher information matrix.

Since asymptotically, $[{\hat{\theta }}_{i}]=\theta_{i},$ the true value of the parameter, then under the null hypothesis, ***S*** *_i_* ~ Normal _*v*_ *_i_* ( **0**, ***I***). I.e. ***S***_*i*_ follows an uncorrelated standard multivariate normal distribution, and 2 log *R _i_* follows a Chi-Squared( *v _i_*) distribution.

The above outline implies that

$$\left(\begin{array}{c} \left({\hat{\theta }}_{1}-\theta_{01}\right)\\ \vdots \\ ({\hat{\theta }}_{K}-\theta_{0K} \end{array}\right)\;∼\;\text{Norma}{\text{l}}_{v_{1}+\cdots +v_{K}}\left(\left(\begin{array}{c} \theta_{1}-\theta_{01}\\ \vdots \\ \theta_{K}-\theta_{0K} \end{array}\right),\left(\begin{array}{ccc} {}[{\hat{\theta }}_{1}] & \cdots & \mathbb{C}[{\hat{\theta }}_{1},{\hat{\theta }}_{K}]\\ \vdots & \ddots & \vdots \\ \mathbb{C}[{\hat{\theta }}_{K},{\hat{\theta }}_{1}] & \cdots & [{\hat{\theta }}_{K}] \end{array}\right)),\;(19\right)$$

where ℂ represents the covariance. Therefore

$$\left(\begin{array}{c} {\text{S}}_{1}\\ \vdots \\ {\text{S}}_{K} \end{array}\right)=\left(\begin{array}{ccc} {\left[{\hat{\theta }}_{1}\right]}^{-1/2} & \cdots & 0\\ \vdots & \ddots & \vdots \\ 0 & \cdots & {\left[{\hat{\theta }}_{K}\right]}^{-1/2} \end{array}\right)\left(\begin{array}{c} \left({\hat{\theta }}_{1}-\theta_{01}\right)\\ \vdots \\ \left({\hat{\theta }}_{K}-\theta_{0K}\right) \end{array}\right)\;(20)$$

$$∼\;\text{Norma}{\text{l}}_{v_{1}+\cdots +v_{K}}\left(\left(\begin{array}{c} {\left[{\hat{\theta }}_{1}\right]}^{-1/2}(\theta_{1}-\theta_{01})\\ \vdots \\ {\left[{\hat{\theta }}_{K}\right]}^{-1/2}(\theta_{K}-\theta_{0K}) \end{array}\right),\left(\begin{array}{ccc} I & \cdots & \text{Cor}[{\hat{\theta }}_{1},{\hat{\theta }}_{K}]\\ \vdots & \ddots & \vdots \\ \text{Cor}[{\hat{\theta }}_{K},{\hat{\theta }}_{1}] & \cdots & I \end{array}\right)\right)\;(21)$$

where $\text{Cor}[{\hat{\theta }}_{i},{\hat{\theta }}_{j}]={\left[{\hat{\theta }}_{i}\right]}^{-1/2}\mathbb{C}[{\hat{\theta }}_{i},{\hat{\theta }}_{j}]{\left[{\hat{\theta }}_{j}\right]}^{-1/2}.$ Thus, ***S*** has a multivariate Normal distribution with variance-covariance matrix equal to the block matrix of individual correlation matrices between the maximum likelihood estimates of each pair of models. Call that matrix ***ρ***. The matrix might be positive-semi-definite, rather than positive-definite, because of collinearity between the *K* models.

When the null hypothesis is true (i.e. *θ*_i_ = *θ*_0__*i*_ ∀ *i* = 1 . . . *K*),

$$SS^{\prime }\;∼\;\text{Wishar}t_{v_{1}+\cdots +v_{K}}(\rho ,1).\;(22)$$

The diagonal of ***SS***′, which has a Wishart-Multivariate-Gamma distribution ^24^, models the dependence within and between the terms in all the sums $2\;\text{log}\;R_{i}=S_{i}^{\prime }S_{i},\;i=1\text{...}K.$ However, the analytical results for this distribution are limited, so in practice Equation 21 is used for simulation. After computing the maximized likelihood ratios *R*_1_, . . . , *R _K_* via Equation 17, the *p*-values are computed from the quantile functions of the corresponding Chi-Squared( *v _i_*) distributions.

### 3.1 Simplified Wishart-Multivariate-Gamma dependence

For the simulations to test the power and false positive rate of the GMP significance thresholds, I used a simplification of the Wishart-Multivariate-Gamma dependence structure with a single parameter, 0 = *ρ* = 1, which measures the strength of positive dependence between the log-likelihood ratios, and hence the *p*-values. I made the simplifyingn assumption that *v _i_* = *v* for all *i* = 1 . . . *K* and (for *i* ≠ *j*)

$$\text{Cor}[{\hat{\theta }}_{i},{\hat{\theta }}_{j}]=\left(\begin{array}{ccc} \rho & \cdots & 0\\ \vdots & \ddots & \vdots \\ 0 & \cdots & \rho \end{array}\right).\;(23)$$

In this scenario, every alternative hypothesis has *v* free parameters compared to its nested null hypothesis. These might be considered to represent parameters that are in some way analogous from test to test. For any pair of likelihood ratio tests, estimates of the analogous parameters are correlated (with correlation coefficient *ρ*) but estimates of non-analogous parameters are uncorrelated. In which case the joint distribution of (2 log *R*_1_, . . . , 2 log *R _K_*)′ when the null hypothesis is true is modelled by the diagonal elements of

$$\text{Wishart}t_{K}\left(\left(\begin{array}{ccc} 1 & \cdots & \rho \\ \vdots & \ddots & \vdots \\ \rho & \cdots & 1 \end{array}\right),v\right).\;(24)$$

In particular, I took *v* = 2, which produces the simple relationship *p _i_* = 1/ *R _i_*. While the parameter *ρ* can be seen as characterizing the strength of the dependence from mild to strong, all models with *ρ* > 0 could be considered as representing a particularly ‘dense’ form of dependence in which every *p*-value is equally correlated with every other one.

## 4 Power-robustness trade-offs

### 4.1 Independence versus arbitrary dependence

To assess the relative performance of the GCLT and RRA significance thresholds for GMPs, I began by directly comparing the thresholds themselves. This allowed me to assess the false positive rates of the RRA threshold under the assumption of independence and the GCLT threshold under worst case dependence.

For a target false positive rate, , the GCLT and RRA significance thresholds for the GMP are written $\Psi_{\text{GCLT},r,K}()$ and ${\text{Ψ}}_{\text{RRA},r,K}()$ respectively. Two quantities can be studied easily:

1. The false positive rate of the RRA threshold under independence:
  $${\text{Ψ}}_{\text{GCLT},r,K}^{–1}\;({\text{Ψ}}_{\text{RRA},r,K}\;())\;(25)$$
2. The false positive rate of the GCLT threshold under worst case dependence (assuming the RRA thresholds are precise):
  $${\text{Ψ}}_{\text{RRA},r,K}^{–1}\;({\text{Ψ}}_{\text{GCLT},r,K}\;())\;(26)$$

The two quantities are expected to be below and above respectively. Neither extreme (independence nor worst case dependence) is thought to represent empirical dependence: Wishart-Multivariate-Gamma dependence scenarios are considered later.

**Figure 1.**
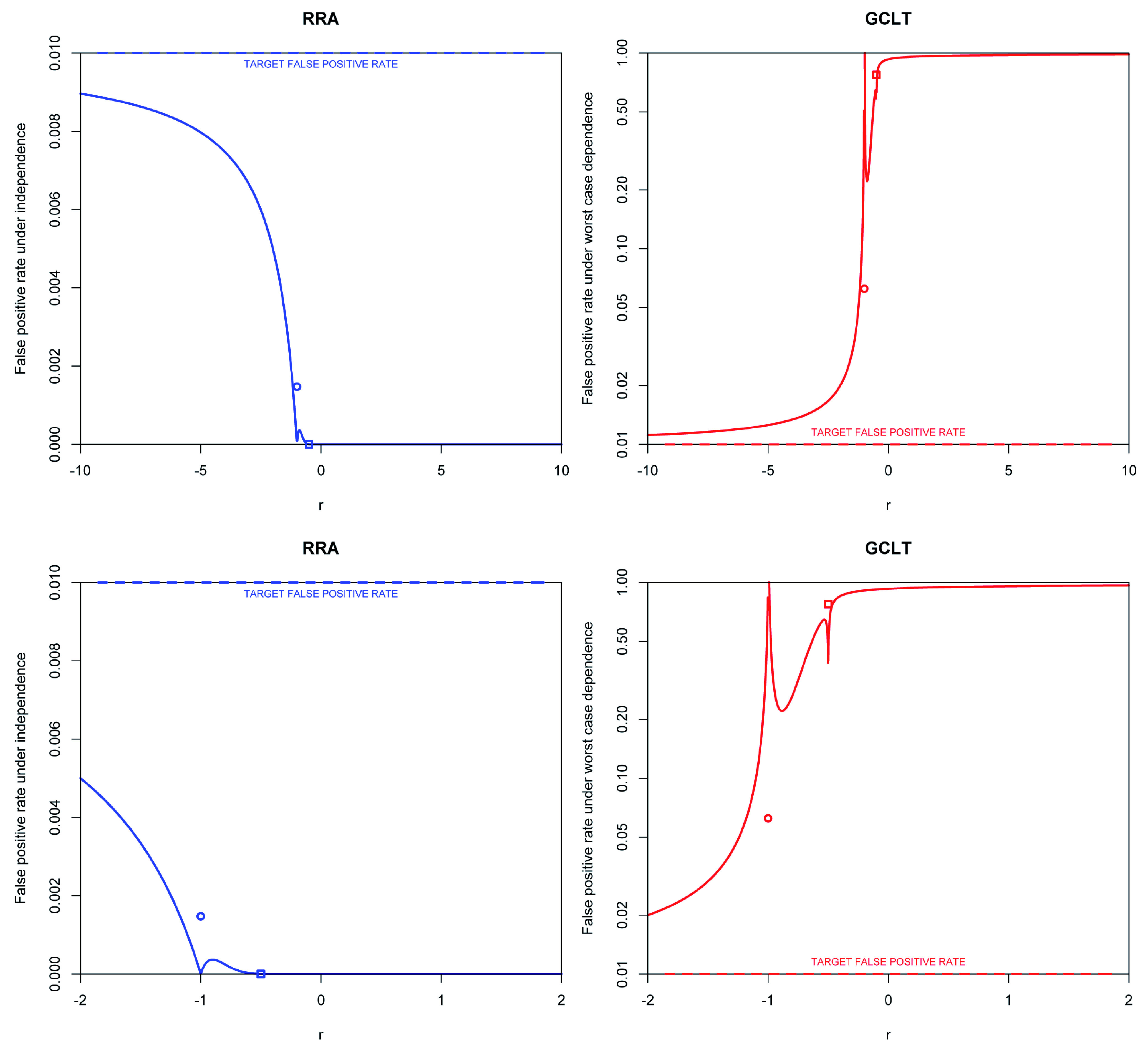
Trade-offs in robustness of GMP significance thresholds to dependence. Two ranges are shown: *r* ∈ [−10, 10] (top panels) and *r* ∈ [−2, 2] (bottom panels). False positive rates using the RRA thresholds assuming independence (left panels, blue lines) and false positive rates using GCLT thresholds assuming worst case dependence and assuming the RRA bounds are precise (right panels, red lines). A target false positive rate of = 0.01 and *K* = 1000 tests were assumed. Discontinuities occur at *r* = −1 (the HMP) and $r=-\frac{1}{2}$. The false positive rates at these positions are marked by a circle and square respectively. Note the logarithmic *y*-axis on the right panels. R code is available as *Extended data* on Figshare: doi:10.6084/m9.figshare.11907033 ^25^.

The two quantities are plotted in Figure 1 for = 0.01 and *K* = 1000 over a range of *r*. The plots support the idea that large negative values of *r* produce tests that are most robust to assumptions regarding dependence and significance thresholds that are most similar. In the extreme case that *r* → –∞, this implies use of Bonferroni and Šidák correction under arbitrary dependence and independence respectively: the resulting significance thresholds converge for small .

Figure 1 also visualises the transition that occurs at $r=–\frac{1}{2}$ between the heavy-tailed ( $r<–\frac{1}{2}$) and light-tailed ( $r\geq –\frac{1}{2}$) distributions of *p ^r^*. Below $r=–\frac{1}{2}$ the heavy tails of the individual *p ^r^* s result in convergence of *M*_*r*, *K*_( *p*_1_, . . . , *p _K_*) ^*r*^ to a Stable distribution, whereas above $r=–\frac{1}{2},$ it converges to a Normal distribution. The Davis-Resnick condition implies that that sum of heavy-tailed random variables is robust to dependence in the tail: Figure 1 (right side) shows that robustness to arbitrary dependence is a process that begins at $r=–\frac{1}{2}$ but requires *r* substantially below $r=–\frac{1}{2}$ to become appreciable. For example, under worst case dependence, the false positive rate of the HMP ( *r* = –1) is still elevated 6.2-fold above the target false positive rate, = 0.01.

As the exponent falls to *r* = –2, the inflation in false positive rate of the GCLT threshold above its target drops to two-fold, a notable value that corresponds to the worst case for *direct interpretation* of the arithmetic mean *p*-value (AMP, *r* = 1) ^26, 27^. If, however, one applies the GCLT significance threshold to the AMP, rather than directly interpreting it, the false positive rate jumps to 0.96 under worst case dependence because direct interpretation of the AMP is highly conservative under independence. The disparity in false positive rates illustrates the vastly superior robustness to dependence of the GCLT threshold, and the GMP in general, at *r* = –2 versus *r* = 1. It also shows that direct interpretation the AMP is questionable: not only is it up to two-fold anti-conservative under worst case dependence between tests, but its power will be highly compromised for independent tests. GMPs with smaller exponents have intrinsically greater robustness to dependence.

Robustness to dependence is a desirable property in the false positive rate, but there may be a cost in terms of the power to reject the null hypothesis when it is false. The well-known conservatism of Bonferroni correction suggests this is inevitable.

### 4.2 Simulations under Wishart-Multivariate-Gamma dependence

For a representative evaluation of test performance it is necessary to consider empirically relevant dependence and to compare not just false positive rates, but power. In this section, I report simulations that I conducted under Wishart-Multivariate-Gamma dependence, a form of dependence motivated earlier by the asymptotic distribution of log-likelihood ratios among dependent tests. I considered a simplified form of Wishart-Multivariate-Gamma dependence in which all tests were equally correlated, with a single correlation parameter *ρ* (defined in section 3.1).

To evaluate false positive rate and power, I considered four scenarios: **Null hypothesis**, **Needle-in-a-haystack**, **Mixture of signals** and **Subtle pervasive signal**. The scenarios differed in the number of *p*-values, out of 1000, simulated under the alternative hypothesis (0, 1, 100 and 1000 respectively), and in the value of the *Z*-statistic $(V{\left[{\hat{\theta }}_{i}\right]}^{-1/2}\left(\theta_{i}\;–\;\theta_{0i}\right);\;$ see section 3) when it was non-zero (n/a, 3.0, 1.25 and 0.7 respectively). The exact values were arbitrary and chosen to produce power ~ 50% under independence. Beside the GMP tests, I conducted Bonferroni, Simes and Fisher tests for comparison. In all cases, I assumed a target false positive rate of = 0.05.

**Figure 2.**
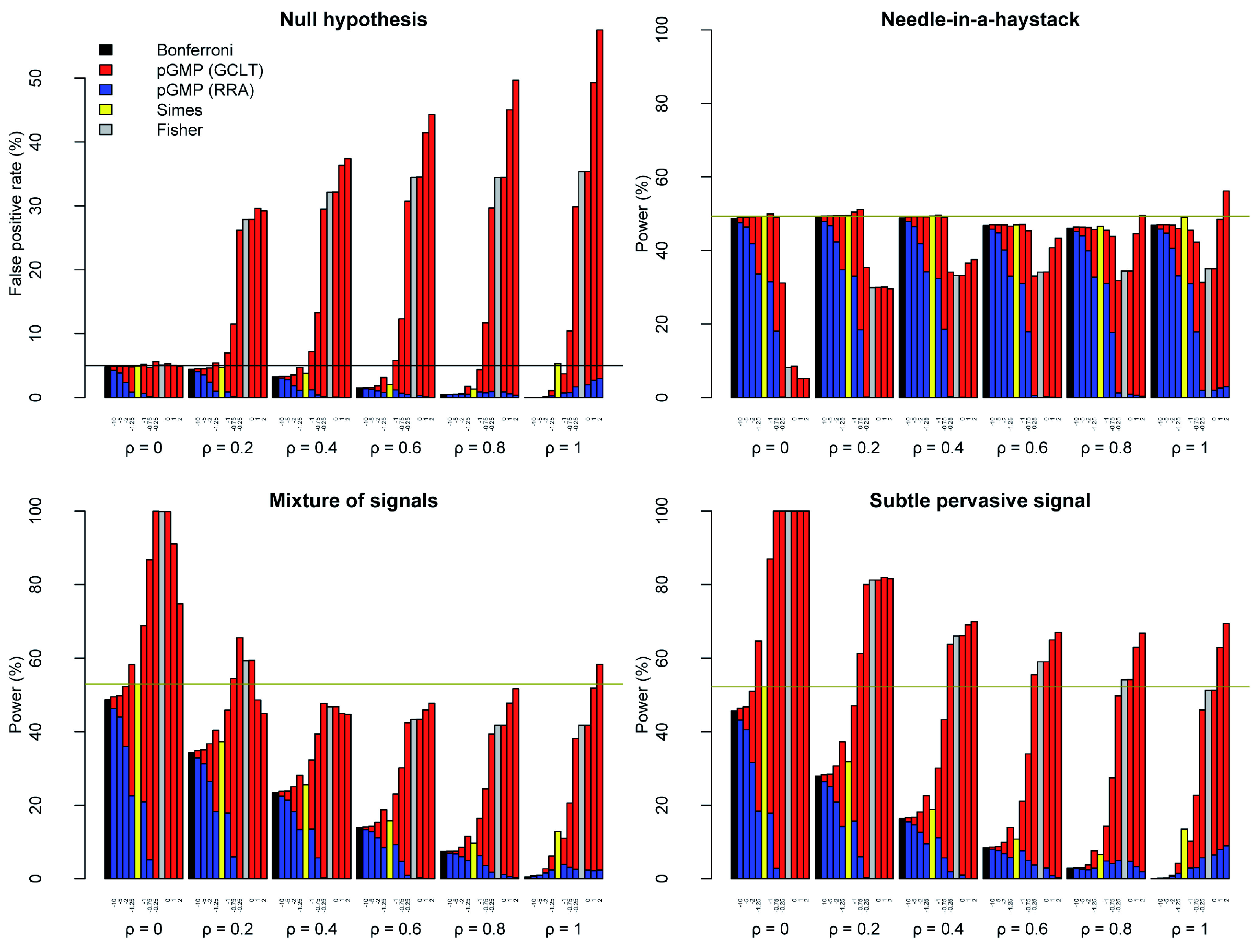
Power-robustness trade-offs for the GMP and related tests. For *r* = {−10, −5, −2, −1.25, −1, −0.75, −0.25, 10 ^−6^, 1, 2} and *ρ* = {0, 0.2, 0.4, 0.6, 0.8, 1}, I conducted 10000 simulations of *K* = 1000 *p*-values under four scenarios (see text). For each scenario, I computed the proportion of simulations in which the GMP was below the significance threshold, calculated by GCLT (red) or RRA (blue). For comparison, I computed the proportion of simulations in which the Bonferroni (black), Simes (yellow) and Fisher (grey) tests were significant. These were plotted next to the GMP they most closely resemble: *r* = −10, −1 and 10 ^−6^ respectively. In all cases, I assumed a target false positive rate of = 5% (horizontal black line). For comparison, the dark yellow horizontal line shows the power of the Simes test. The R code is available as *Extended data* from Figshare: doi:10.6084/m9.figshare.11907033 ^25^.

***False positive rates.*** Using the GCLT thresholds, the GMP with *r* < –1 exhibited false positive rates that were close to the target of = 5% under mild dependence ( *ρ* ≤ 0.2) and substantially below it under strong dependence ( *ρ* ≥ 0.6) ( Figure 2). The GMP with *r* > –1 exhibited substantial inflation of false positive rates under mild to strong dependence. In contrast, the HMP ( *r* = –1) maintained a false positive rate close to the target in all cases, erring on the side of inflation for 0.2 *≤ ρ ≤* 0.6, as observed previously ^17, 19^.

Using the RRA thresholds, the false positive rate of the GMP was always well-controlled below the target of = 5%, as expected, and therefore below the rates achieved using the GCLT thresholds. Often it was far below the target false positive rate. Usually it was even below the false positive rate of the Bonferroni procedure ( Figure 2).

***Power.*** In considering power, the GCLT thresholds for *r* > –1 can be disregarded as inadmissible when *ρ* ≠ 0 because they could not control the false positive rate close to the target. In this range, the RRA thresholds were admissible but exhibited very poor power under all scenarios ( Figure 2). The conclusion that neither the GCLT nor RRA thresholds are useful in the range –1 < *r* < –1/2 is relevant to the later section on the interpretation of the GMP ( section 6). From a practical perspective, these simulations suggest that GMPs with *r > −*1, including the geometric mean *p*-value ( *r* = 0; Fisher’s method) and the arithmetic mean *p*-value ( *r* = 1), are not useful unless independence or an explicit model of dependence can be safely assumed, e.g. 7, 16.

**Figure 3.**
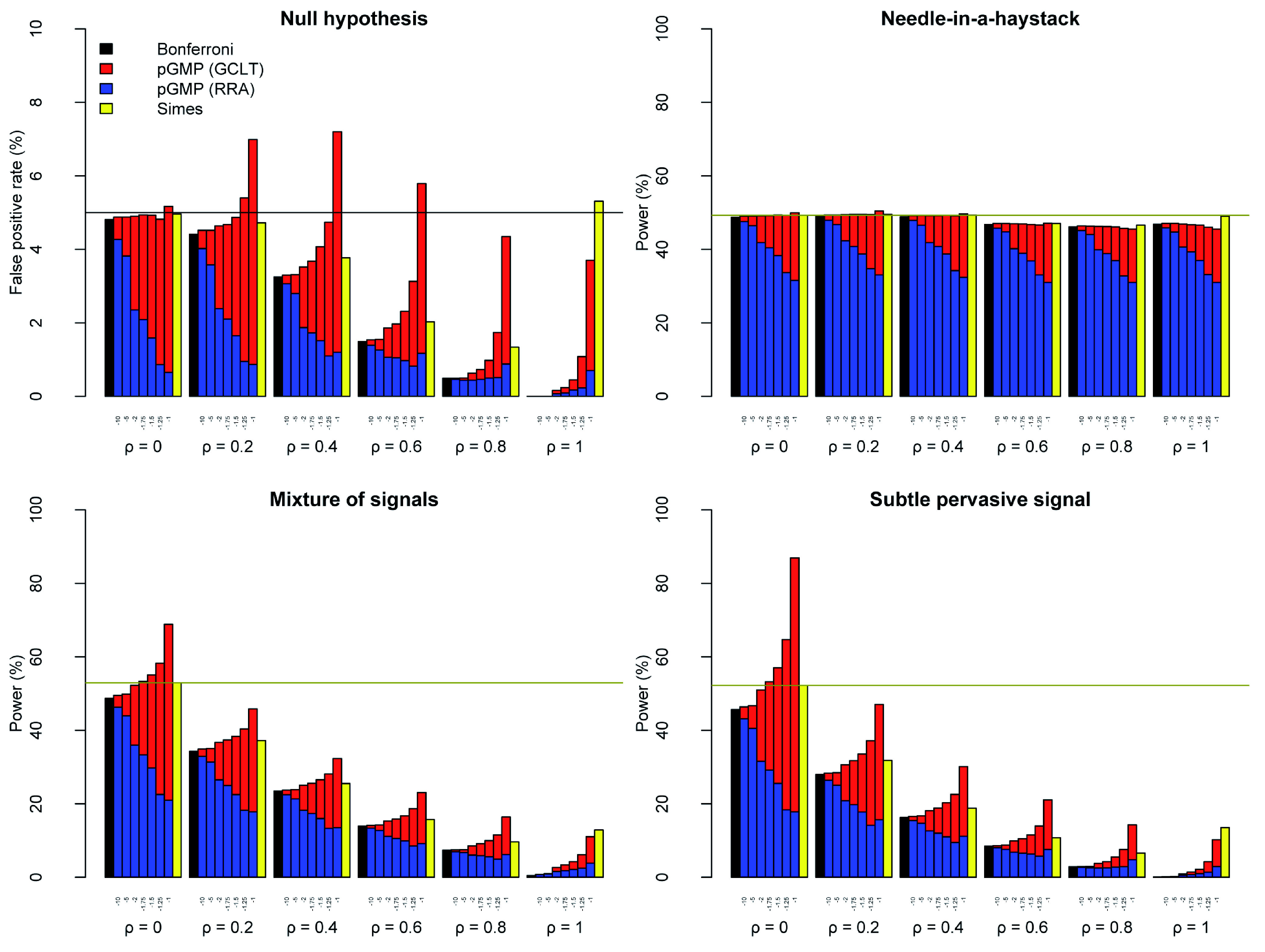
Power-robustness trade-offs for the GMP and related tests. As for Figure 2 but considering GMP with *r* = {−10, −5, −2, −1.75, −1.5, −1.25, −1}, i.e. only very heavy-tailed distributions. R code is available as *Extended data* on Figshare: doi:10.6084/m9.figshare.11907033 ^25^.

In the Needle-in-a-haystack scenario, the GCLT thresholds for *r* ≤ –1 achieved power comparable to, or slightly worse than, the Bonferroni procedure. For –10 ≤ *r* ≤ –1, the RRA thresholds were worse than the Bonferroni procedure, considerably so for –2 ≤ *r* ≤ –1 ( Figure 3).

In both the Mixture of signals and Subtle pervasive signal scenarios, the power of the GCLT thresholds increased from *r* = –10 to *r* = –1. The trend was reversed for the RRA thresholds (except for *ρ* = 1). The power of the RRA thresholds was uniformly worse (often substantially worse) than for the GCLT thresholds, and it was usually worse than for the Bonferroni thresholds too. The effect of increasing the strength of dependence was to reduce power for the GCLT, RRA and Bonferroni thresholds.

The Simes test resembles the HMP using the GCLT threshold, both in terms of interpretation and performance ^9^. Simes’ test had the advantage of avoiding inflation in false positive rate under the Null hypothesis with 0.2 ≤ *ρ* ≤ 0.6. The power of Simes’ test was no better than the HMP (except at *ρ* = 1), but often it was appreciably worse in the Mixture of signals and Subtle pervasive signal scenarios. For smaller values of *r*, e.g. *r* = –1.25, the inflation in false positive rate under mild dependence was reduced compared to the HMP. The GCLT threshold at *r* = –1.25 also generally outperformed Simes’ test in terms of power, with the exception of *ρ* = 1. No value of *r* enabled the RRA thresholds to outperform the power of Simes’ test.

As expected, Bonferroni correction behaved much like the GMP with *r* = –10 under both the GCLT and RRA thresholds. As expected, Fisher’s method was indistinguishable from the GMP with *r* = 10 ^–6^ under the GCLT threshold. For *ρ* = 0, Fisher’s method could not be bettered in the Mixture of signals and Subtle pervasive signal scenarios, but it was roundly outperformed in the Needle-in-a-haystack scenario ( Figure 2).

In conclusion, the HMP (with GCLT threshold) and Simes’ test appear to offer superior performance to the alternatives over the range of dependence structures considered. The HMP enjoys greater power than Simes’ test, at the cost of an inflated false positive rate. For end-users, the relative importance of power versus a conservative false positive rate will influence the choice of test. The simulations support the claim that the HMP is robust to dependence in the sense that the realized false positive rate is close to the target across all dependence structures investigated.

### 4.3 Inherent power of the HMP vs Simes’ test

**Figure 4.**
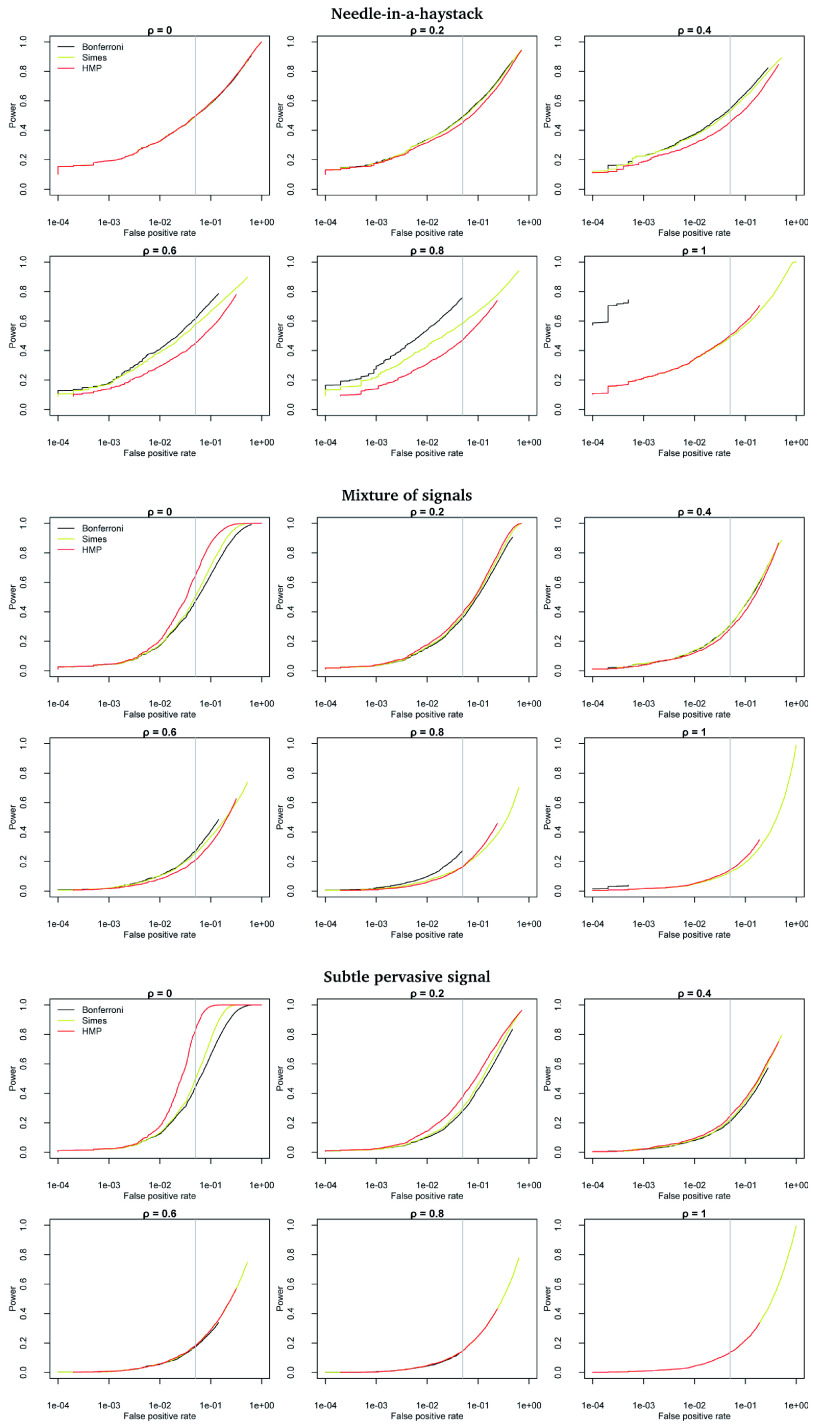
Receiver-operator curves (power vs false positive rate) by strength of dependence and scenario. R code is available as *Extended data* on Figshare: doi:10.6084/m9.figshare.11907033 ^25^.

The superior power of the HMP compared to Simes’ test in the presence of dependence is attributable, in part, to its higher false positive rate. However, receiver-operator curves (ROCs, Figure 4) summarizing the simulations above show that in the Mixture of signals and Subtle pervasive signal scenarios, the HMP is inherently more powerful than Simes’ test. However, the advantage is reduced, and even reversed, as dependence increases. Simes’ test is inherently more powerful than the HMP in the Needle-in-a-haystack scenario.

**Figure 5.**
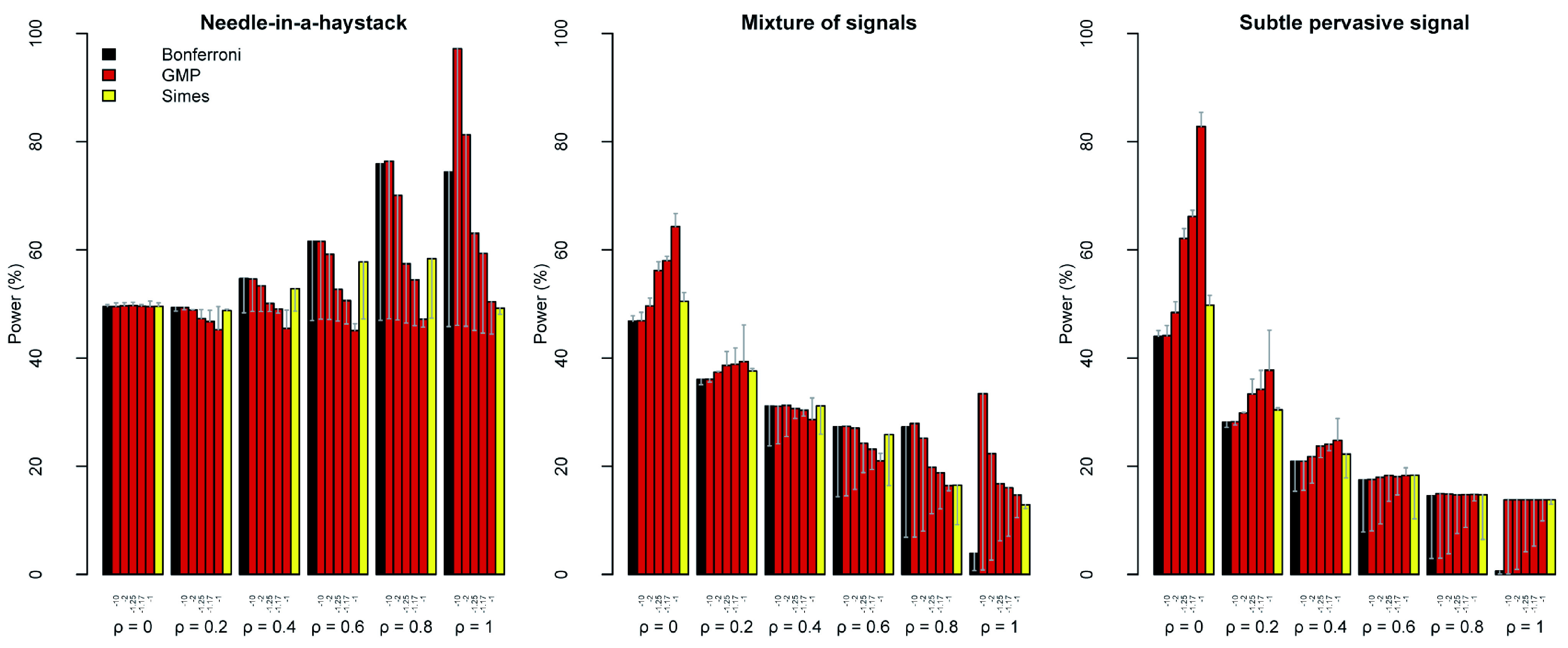
Inherent versus actual power for the Bonferroni, GMP and Simes tests. GCLT thresholds were used for the GMPs, with *r* = {−10, −2, −1.25, −1.17, −1}. Actual power (grey whiskers) exceeded the inherent power (bars) when the false positive rate was inflated relative to its target of = 5%. R code is available on Figshare: doi:10.6084/m9.figshare.11907033 ^25^.

Inherent power (the power of a test when its threshold achieves the target false positive rate) cannot be realized without analytical results, which are not available for Wishart-Multivariate-Gamma dependence, or simulations. Nevertheless, one can use simulations to compare inherent power to actual power to quantify the shortfall or excess power attributable to conservatism or anti-conservatism of the false positive rate. In Figure 5, the bars show inherent power while the grey whiskers compare that to actual power, using GCLT thresholds. When the grey whisker exceeded the bar, as it often did for the HMP ( *r* = –1), the actual power was elevated relative to inherent power because of an inflated false positive rate. When the grey whisker fell below the bar, as it usually did for Simes’ test, the actual power was less than the inherent power because of a conservative false positive rate.

For *r* < –1, the tendency for actual power to exceed inherent power was reduced, and often reversed compared to the HMP. However, the cost of this greater robustness to dependence was reduced actual power in the Mixture of signals and Subtle pervasive signal scenarios. Empirically, the trend appeared to be monotonic except for when log *K*/(1 – log *K*) < *r* < –1, in which region the inherent and actual power were drastically reduced (not shown). Therefore, *r* = log *K*/(1 – log *K*), which equalled –1.17 for *K* = 1000, was the GMP with *r* < –1 whose characteristics most closely resembled the HMP. This coincided with the derivation of the RRA threshold for *r* = –1, which was taken as the tightest bound based on *r* < –1, which occurs at *r* = log *K*/(1 – log *K*) ^15^. Whether this recommends the use of the GMP at *r* = log *K*/(1 – log *K*) over the HMP is unclear. Figure 3 showed that GMPs with *r* < –1 can still be subject to mildly inflated false positive rates using GCLT thresholds, even if they are attenuated relative to the HMP. Bonferroni usually over-powered the GMP using RRA thresholds.

## 5 Strong-sense family-wise error rates

One of the advantages of the HMP procedure is its ability to test arbitrary combinations of the *K p*-values while controlling the strong-sense familywise error rate at a pre-specified level , known as multilevel testing ^9^. A full assessment of the relative performance of other GMPs to the HMP therefore involves a comparison of their performance as multilevel tests. This requires a closed testing procedure (CTP ^14^) to be derived for the GMP with any exponent *r*.

Suppose that ℜ is an index set of the *i* = 1 . . . *K p*-values. As shorthand, write

$${\bar{p}}_{r,ℜ}=M_{r,\left|ℜ\right|}\left(\left\{p_{i}:iℜ\right\}\right)\;(27a)$$

$${\bar{p}}_{r}=M_{r,K}\left(p_{1},\ldots ,p_{k}\right).\;(27b)$$

To define the closed testing procedure of the multilevel test, find the least stringent (i.e. largest) value below 1 of the factor *f*_|ℜ|_ for which the following condition

$${\bar{p}}_{r,ℜ}\leq f_{|ℜ|}\Psi_{r,|ℜ|}\left(\right)\;(28)$$

(interpreted as significance of subset ℜ) implies the significance of all tests combined, i.e.

$${\bar{p}}_{r}\leq \Psi_{r,K}\left(\right).\;(29)$$

Since

$${\bar{p}}_{r}^{r}=\frac{\left|ℜ\right|}{K}{\bar{p}}_{r,ℜ}^{r}+\frac{\left|{ℜ}^{\prime }\right|}{K}{\bar{p}}_{r,{ℜ}^{\prime }}^{r}\;(30)$$

then Equation 28 implies that

$${\bar{p}}_{r}\leq {\left(\frac{\left|ℜ\right|}{K}f_{\left|ℜ\right|}^{r}\Psi_{r,\left|ℜ\right|}{\left(\right)}^{r}+\left(1-\frac{\left|ℜ\right|}{K}\right)\right)}^{1/r}\;(31)$$

assuming the least favourable case that ${\bar{p}}_{r,{ℜ}^{\prime }}=1.$ The condition in Equation 29 is therefore satisfied by

$$f_{\left|ℜ\right|}=\min\left\{1,{\left(\frac{\Psi_{r,K}{\left(\right)}^{r}-\left(1-\frac{\left|ℜ\right|}{K}\right)}{\frac{\left|ℜ\right|}{K}\Psi_{r,\left|ℜ\right|}{\left(\right)}^{r}}\right)}^{1/r}\right\}.\;(32)$$

The above reveals that the multilevel test suffers complications when *r* > 0. This is because for subsets of *p*-values smaller than *K*(1 − Ψ _*r*, *K*_ () ^*r*^), the numerator of Equation 32 can be negative when *r >* 0. The interpretation is that there is no value of ${\bar{p}}_{r,ℜ}$ small enough to compensate for the assumption that ${\bar{p}}_{r,{ℜ}^{\prime }}=1.$ Thus no individual *p*-value can be sufficiently significant to guarantee that the combined test is significant. (Although knowledge of the rank of the *p*-value would alter this conclusion, e.g. knowing it was the maximum. The problem arises because this multilevel test is a shortcut to the full CTP and is based on single *p*-values. CTPs are not ruled out for *r >* 0 in general.) From a practical perspective, it means that when *r >* 0, no subsets of *p*-values smaller than *K*(1 − Ψ _*r*, *K*_ () ^*r*^) can be significant within this multilevel test, limiting the finest levels at which inference can be made.

The multilevel test simplifies when *r* < –1 because the GCLT thresholds ( Equation 14 and Table 1) and RRA thresholds ( Table 2) possess the property that Ψ *_r,K_*()/Ψ _*r*,|ℜ|_()=(|ℜ|/ *K*) ^1+1/ *r*^. This allows a more stringent form of Equation 32 to be expressed as

$$\begin{array}{l} f_{\left|ℜ\right|}=\min\;\left\{1,{\left(\frac{\Psi_{r,K}{\left(\right)}^{r}}{\frac{\left|ℜ\right|}{K}\Psi_{r,\left|ℜ\right|}{\left(\right)}^{r}}\right)}^{1/r}\right\},\;r<0\\ \;=\frac{\left|ℜ\right|}{K},\;r<-1. \end{array}\;(33)$$

This produces a convenient form of the CTP:

$${\bar{p}}_{r,ℜ}\leq \left(\frac{\left|ℜ\right|}{K}\right)\;\Psi_{r,\left|ℜ\right|}\left(\right)={\left(\frac{\left|ℜ\right|}{K}\right)}^{-1/r}\;\Psi_{r,K}\left(\right),\;r<-1.\;(34)$$

Thus by RRA one has,

$${\bar{p}}_{r,ℜ}\;\leq \;\frac{{\left|ℜ\right|}^{-1/r}}{K}\frac{r+1}{r},\;r<-1\;(35)$$

and by GCLT ( Equation 16 and 9), one has a simple small approximation

$${\bar{p}}_{r,ℜ}\;\leq \;\frac{{\left|ℜ\right|}^{-1/r}}{K},\;r\leq -1,\;\to 0.\;(36)$$

Further, Equation 34 shows that all CTPs in the range −∞ < *r* < −1 have a cost relative to Bonferroni ( *r* → −∞) in the sense that the significance threshold for an individual *p*-value is more stringent by a factor $\frac{r+1}{r}$ (by RRA) or ${}^{-1}{\left(C_{-1/r}F_{-1/r,1}^{-1}\left(\right)\right)}^{1/r}$ (by GCLT), although Equation 36 shows that the latter is close to 1 for small . Intuitively, this represents the cost of the additional power to make statements about *groups* of *p*-values over and above the statements one can make about *individual p*-values ^17^. However, the multilevel Simes test does not incur this penalty.

## 6 Interpretation

The *p*-value can be seen as a low-dimensional summary of the data that is relevant to hypothesis testing. From this perspective, the distribution of the *p*-value under the alternative can be modelled directly, e.g. 28. Heard and Rubin-Delanchy ^4^ considered Beta distributions for the *p*-value under the alternative,

$$p_{i}|{ℳ}_{i}∼\text{Beta}\left(\xi ,\zeta \right),$$

subject to the constraint that the density is non-increasing in *p*, which implies that 0 *< ξ ≤* 1 and 1 *≤ ζ*. By the Neyman-Pearson lemma ^29^, they argued that one can identify uniformly most powerful tests for combining independent *p*-values under the Beta distribution assumption. Fisher’s method was optimal for *ζ* = 1, the subset of Beta distributions that have been advocated for local alternatives ^30^. Pearson’s method ^31^ was optimal for *ξ* = 1, the subset of Beta distributions that have been advocated for simple alternatives ^30^.

The likelihood ratio for a *p*-value that is Beta distributed under the alternative can be written

$${\text{BF}}_{i}\;=\;\frac{p_{i}^{\xi -1}{\left(1-p_{i}\right)}^{\zeta -1}}{\text{B}\left(\xi ,\zeta \right)},\;(37)$$

where B( *·*) is the Beta function. The notation BF _*i*_ is used because the local alternatives assumption amounts to a Bayesian prior distribution over effect sizes with hyper-parameter *ξ*, and the likelihood ratio is therefore a Bayes factor.

A mean Bayes factor (or likelihood ratio) of the form

$$\bar{\mathrm{BF}}\;=\;\frac{1}{K}\sum_{i=1}^{K}\frac{p_{i}^{\xi -1}{\left(1-p_{i}\right)}^{\zeta -1}}{\text{B}\left(\xi \text{,}\zeta \right)}\;(38)$$

arises in a model-averaging setting in which the alternative hypothesis is a mixture of individual, mutually exclusive alternatives. Here each *p*-value uses the *same* data to evaluate each competing alternative hypotheses against a common nested null hypothesis. This implies an interpretation of GMPs as uniformly most powerful tests for model-averaged alternative hypotheses, each of which is a different local alternative to the common nested null hypothesis. Under these conditions, *ξ* = 1 + *r* and *ζ* = 1 so that

$$M_{r,K}{\left(p_{1},\ldots \;,p_{K}\right)}^{r}\;=\;\bar{\text{BF}}=\frac{1}{K}\sum_{i=1}^{K}(1+r)p_{i}^{r}.\;(39)$$

The model-averaging interpretation applies when *−*1 *< r <* 0. Unfortunately, the simulations summarized in Figure 2 showed that for *−*1 *< r <* 0, the GCLT threshold suffered greater elevation in false positive rates than the HMP. (The RRA threshold is not considered here because power was low and usually worse than Bonferroni). Nevertheless, outside the range *−*1 *< r <* 0, the GMP can be viewed as a bound on the model-averaged Bayes factor. Defining *r*⋆ < *r* < 0, one has the relationship

$$\bar{\text{BF}}\;\leq \;(1+r)M_{r⋆,K}{\left(p_{1},\ldots \;,p_{K}\right)}^{r}\;(40)$$

because *M*_*r*, *K*_ ( *p*_1_, . . . , *p*_*K*_) ≥ *M*_*r⋆*, *K*_ ( *p*_1_, . . . , *p*_*K*_). Therefore GMPs supply upper bounds on Bayes factors when *r ≤ −*1 and lower bounds on Bayes factors when *r >* 0. The HMP supplies the tightest upper bound for well-powered tests ( *r ↓ −*1). Simes’ test and Bonferroni can be framed similarly as providing lower bounds on the model-averaged Bayes factor.

While the HMP has a natural interpretation as a tight bound on the model-averaged Bayes factor for well-powered tests, it does not correspond exactly to any Bayes factor and it does not control the frequentist false positive rate exactly for some dependence structures, making it an approximation to both approaches that could be criticized for satisfying neither.

## 7 Conclusions

**Figure 6.**
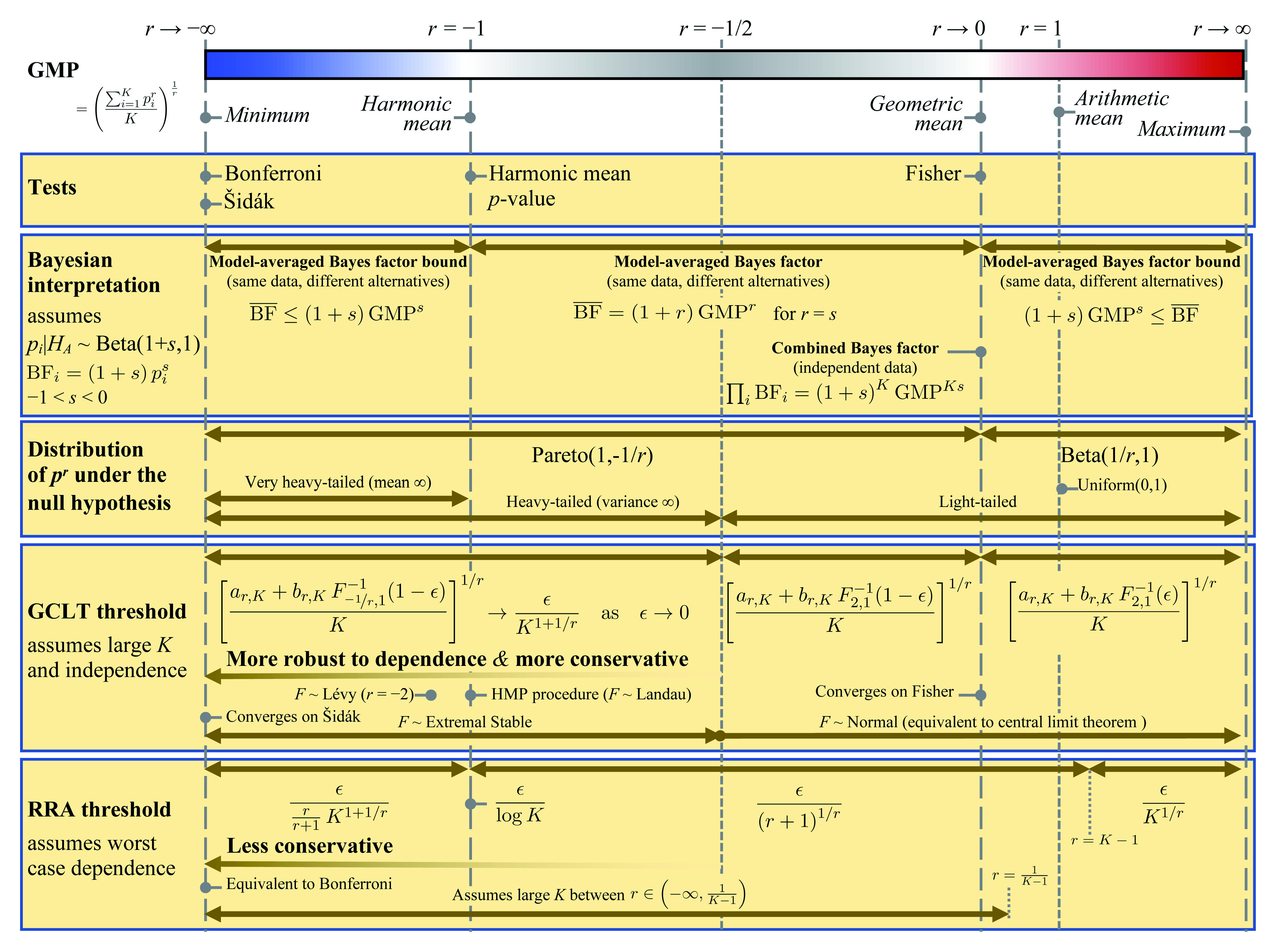
Summary of the generalized mean *p*-value (GMP), its relation to other tests, Bayesian interpretation, significance thresholds derived using generalized central limit theorem (GCLT) and robust risk analysis (RRA) ^15^, test assumptions and performance characteristics.

Taking the generalized mean *p*-value of a group of tests extends a number of existing methods for combining *p*-values including the Bonferroni, Šidák, harmonic mean *p*-value and Fisher procedures ^7, 9– 12^ ( Figure 6). The interpretation varies by (i) the exponent of the GMP, and (ii) the key assumption regarding dependence between the tests. Two appealing interpretations occur when *−*1 *< r <* 0 and *r* = 0. When *−*1 *< r <* 0, combining *p*-values using the GMP can be interpreted as model averaging if the same data have been used to evaluate mutually exclusive alternative hypotheses against a common null hypothesis. In this interpretation, when *r* is closer to *−*1, very small *p*-values are assumed more likely when the alternative hypothesis is true, implying the individual tests were more powerful. Outside the range *−*1 *< r <* 0, the GMP is interpretable as approximating this approach, with *r* = *−*1 (the HMP) offering the closest approximation for well-powered tests. When *r* = 0, combining *p*-values using the GMP can be interpreted as aggregating evidence for related pairs of alternative and null hypotheses, if independent data were used for the individual tests, in which case the method is equivalent to Fisher’s for many tests. Outside these specific interpretations, the GMP offers a flexible non-parametric approach to combining *p*-values where *r* controls the sensitivity to small values.

GMPs are not directly intepretable as *p*-values in general ^15^. Instead, significance thresholds are required. Generalized central limit theorem and robust risk analysis provide convenient methods for defining significance thresholds for GMPs that do not require explicit knowledge of the dependence structure, providing robustness to dependence to varying degrees. RRA thresholds provide robustness to arbitrary dependence in the sense that the false positive rate will not exceed the target ^15^. GCLT thresholds provide a weaker form of robustness to forms of dependence that satisfy the Davis-Resnick condition ( Equation 6), but only for sufficiently small values of the target false positive rate and *r ≤ −*1. Subject to these conditions, the HMP is the only GMP that can be directly interpreted as if it were a *p*-value ^9, 17^.

The cost of robustness to arbitrary dependence was too high to make the RRA thresholds directly useful in practice, because they were usually rendered less powerful than the Bonferroni procedure in simulations ( Figure 2, Figure 3). However, they remain theoretically valuable because they bound the worst-case inflation of the false positive rate of the GCLT thresholds. The RRA and GCLT thresholds agreed more closely as *r → −∞*. The trend for RRA thresholds to deliver less powerful tests as *r* increased was reversed for GCLT thresholds. In practice the GCLT thresholds were generally more powerful than Bonferroni, and increasingly so as *r* increased, but they began to suffer inflated false positive rates. The GCLT threshold for the HMP has previously been shown to suffer modest inflation under mild dependence ^19^. However, above *r* = *−*1, the point at which the underlying distribution of *p* ^*r*^ transitioned from very heavy tailed ( *r ≤ −*1) to heavy tailed ( *−*1 *< r ≤ −*1 */*2), inflation accelerated to the point that there was no useful robustness to non-independence ( Figure 2). Despite this problem, incorporating knowledge of dependence into standard central limit theorem, applicable for light-tailed distributions ( *r > −*1 */*2), would be straightforward, requiring knowledge only of $\text{ℂ}\left(P_{i}^{r},P_{j}^{r}\right)$. The loss of robustness to dependence recommends against the use of the GMP with GCLT thresholds for *r > −*1 except when independence can be safely assumed.

The arithmetic mean *p*-value, which arises in numerous applications including posterior predictive *p*-values, is known to be directly interpretable subject to a maximum two-fold inflation in false positive rate ^25, 27^. However, for the dependence structures considered here, there appeared to be little merit in *direct interpretation* of the AMP: to do so would be far too conservative under independence and likely less powerful than Bonferroni under worst-case dependence. An interesting alternative might be the GMP with *r* = *−*2, whose GCLT threshold is $/\sqrt{K}$ for small and does suffer at worst two-fold inflation under arbitrary dependence (for large *K*). Unlike the HMP, the GMP with *r* = *−*2 did not suffer even mild inflation in false positive rate in simulations, but the HMP was more powerful ( Figure 3). The GMP with *r* = *−*2 performed remarkably similarly in false positive rate and power to Simes’ test ^20^. Simes’ test and the HMP can be seen as offering similarly-performing but complementary solutions to the power-robustness trade-off for model-averaged *p*-values ^9^, erring on the side of conservatism versus power respectively.

There were several limitations in the current study: (i) Equal weights were assumed throughout, although simulations for the HMP ^9^ suggest there may be robustness to unequal weights, at least for *r ≤ −*1. (ii) The distribution of *p*-values under the null hypothesis was assumed to be Uniform(0,1). However, valid *p*-values are generally defined such that Pr( *p* < *x*| ℳ _0_) ≤ *x*. Conservatism of this sort was not explored, but is likely to profoundly diminish the power of the GMP. (iii) The simulations considered here assumed a particular form of dependence in which the *p*-values were chi-squared tail probabilities of underlying log-likelihood ratios that for large samples would follow a Wishart-Multivariate-Gamma distribution. A particularly dense form of dependence was assumed that applied to every pair of *p*-values. Some results, such as the inflation in false positive rates for the GCLT thresholds in the region *r* = *−*1, will depend quantitatively on the details of the simulations. The conclusion that the RRA thresholds are less powerful than Bonferroni may apply more widely because it stems from the theoretical divergence in GCLT and RRA thresholds as *r* increases, and it might seem reasonable to assume that the behaviour of empirically relevant *p*-value dependence is intermediate between their respective assumptions of independence and arbitrary dependence.

In conclusion, simulations under a form of dependence relevant to *p*-values calculated from likelihood ratio tests showed that the GMP is practically useful for combining dependent *p*-values for exponents *r* ≤ −1 using thresholds derived from generalized central limit theorem. Robust risk analysis provides corresponding upper bounds on the false positive rate under worst case dependence ^15^, but these upper bounds were not directly useful as significance thresholds because they produced tests typically less powerful than Bonferroni. Those wishing to protect themselves against worst case dependence should therefore prefer the Bonferroni procedure. However, there is increasing interest in exploiting heavy tail behaviour to confer desirable properties in terms of power and robustness to dependence upon combined tests ^9, 32^, and the GMP for *r* ≤ −1 with GCLT thresholds extends this class of methods.

## 8 Data availability

### Underlying data

No underlying data are associated with this article.

### Extended data

Figshare: R code for Figures. https://doi.org/10.6084/m9.figshare.11907033.v1 ^25^.

This project contains R code for Figure 1– Figure 5.

Extended data are available under the terms of the Creative Commons Attribution 4.0 International license(CC-BY 4.0).
