# Supplementary material for: Generalized mean p-values for combining dependent tests: comparison of generalized central limit theorem and robust risk analysis
Source: Wellcome Open Res. 2020 Mar 31;5:55. [Version 1] doi: 10.12688/wellcomeopenres.15761.1 (PMC7836035; doi:10.12688/wellcomeopenres.15761.1)
Supplement: Peer review history

# Peer review: Reviewer response for version 1

**DOI:** 10.21956/wellcomeopenres.17286.r41937

**Published:** 2021-01-22

**Authors:** Xuexia Wang

**Affiliations:** 1 Department of Mathematics, University of North Texas, Denton, TX, USA

Methods of combining p-values are very useful in many fields, such as genetic meta analysis by combining multiple studies and gene-based genetic association studies by combing multiple single marker tests together. Several combination test statistics underpinning the Bonferroni, Šidák, harmonic mean p-value (HMP), Fisher, and other procedures are special cases of the generalized mean p-value (GMP). GMP cannot be interpreted directly as p-values since they are not uniformly distributed under the null hypothesis, but the GMP can be used as a test statistic by calculating a significance threshold for rejecting the null hypothesis with a pre-specified limit (significance level). In this manuscript, the author considered significance thresholds for the GMP and compared their performance under different dependence assumptions. The author performed very important work and generated some very valuable results.

 There are some published work regarding to the significance thresholds. Vovk and Wang (Vovk, 2018) ^1^ derived significance thresholds for GMPs under arbitrary dependence using robust risk analysis (RRA). Wilson (Wilson, 2019) ^2^ derived a considerably less stringent threshold using generalized central limit theorem (GCLT). In this manuscript, the author further derived significance thresholds for GMPs using GCLT and motivated a model of p-value dependence based on the Wishart-Multivariate-Gamma distribution. In simulation studies, the author simulated data under the model to test the power and false positive rates of the GCLT and RRA significance thresholds. Simulations under a form of dependence relevant to p-values calculated from likelihood ratio tests showed that the GMP is practically useful for combining dependent p-values for exponents r ≤ −1 using thresholds derived from GCLT. The RRA thresholds produced tests that were usually less powerful than Bonferroni, while the GCLT thresholds produced tests more powerful than Bonferroni, for all r> − ∞.

 In general, this is a very important work. I have several minor comments:

1. In the Introduction, the author mentioned “combining Z-statistics, rather than p-values, allows parameters to be jointly estimated across datasets”. What parameters can be jointly estimated across datasets?
2. Below Figure 2, the author mentioned “I conducted 10000 simulations”. Does the 10000 simulations mean 10000 replications?
3. It is not very clear how the author simulates data under the three scenarios: Needle-in-a- haystack, Mixture of signals and Subtle pervasive signal (4.2, page 10). How did you simulate the p-values considering dependence following the Wishart-Multivariate-Gamma distribution for each scenarios?
4. There are some typos or grammar errors where I have highlighted in the manuscript.

Is the work clearly and accurately presented and does it cite the current literature?

Yes

If applicable, is the statistical analysis and its interpretation appropriate?

Yes

Are all the source data underlying the results available to ensure full reproducibility?

Yes

Is the study design appropriate and is the work technically sound?

Yes

Are the conclusions drawn adequately supported by the results?

Yes

Are sufficient details of methods and analysis provided to allow replication by others?

Yes

Reviewer Expertise:

Biostatistics, statistics, statistical genetics

I confirm that I have read this submission and believe that I have an appropriate level of expertise to confirm that it is of an acceptable scientific standard.

## References

[1] : Combining P-Values Via Averaging. *SSRN Electronic Journal*.2018; 10.2139/ssrn.3166304 10.2139/ssrn.3166304

[2] : The harmonic meanp -value for combining dependent tests. *Proceedings of the National Academy of Sciences*.2019;116(4) : 10.1073/pnas.1814092116 1195-1200 10.1073/pnas.1814092116 PMC634771830610179

# Peer review: Reviewer response for version 1

**DOI:** 10.21956/wellcomeopenres.17286.r41707

**Published:** 2020-12-18

**Authors:** Raif Rustamov

**Affiliations:** 1 Data Science and AI Research, Chief Data Office, AT&T Inc, Bedminster, NJ, USA

P-value combination approaches have started attracting increasing attention after the recent introduction of the Harmonic Mean P-value (HMP) ^1^ and Cauchy Combination Test ^2^ combination methods. The motivations for these two approaches are very different - Bayesian and Cauchy additive property, but interestingly, these methods are asymptotically equivalent in the most relevant regime, namely for small p-values ^3^. A fundamental question is whether there are other p-value combination approaches that a) have a different asymptotic behavior and b) are at least as efficient as these two methods. The paper under review analyzes this problem from both theoretical and simulation aspects, and provides evidence that such a method may not exist.

 The main theoretical contribution of this paper is to generalize the logic behind HMP to a parametrized class of p-value combination methods by computing the appropriate thresholds via generalized central limit theorem (GCLT). These thresholds are based on the independence assumption yet are robust to violations. They are directly useful for practical applications, and provide an alternative to the robust risk analysis (RRA) based thresholds ^4^ that are universal but rather conservative. An argument using a result from Davis-Resnick shows that there exists an explicit condition that guarantees the proper test size control at the limit. It seems to me that this condition can be relaxed; for example a theorem in ^2^ shows that a certain bivariate normality condition leads to the correct limiting size control. I take this as the evidence that the proposed thresholds can be used more widely.

 It is the comparison of GCLT and RRA thresholds that is very revealing. It becomes clear that HMP, which corresponds to the case of r=-1 has the most potential for gains due to the GCLT/RRA threshold ratio being log K. For methods with r < -1 the thresholds differ at most by a factor of 2 and there is little room to provide viable alternatives to HMP. Finally, methods with r > -1 are neither robust to dependencies (as shown by experiments) nor provide threshold gains at the limit of large K. This can be seen as evidence to the unique place of r=-1 and that the only interesting/efficient methods for p-value combination should have the same asymptotic behavior as HMP. Of course, this only establishes the asymptotic form of the tail, but the specific form can make big difference in practice.

 The paper continues on to experimentally compare this class of methods using both GCLT and RRA thresholds for different values of r. The experiments consider a wide variety of setups and are rather detailed. One aspect of these experiments is that the proposed setup is very challenging due to the inclusion of all-pair dependencies, and it essentially pushes this methodology to the limit. As a consequence, the presented results may seem somewhat discouraging even for HMP. However, in my own work ^3^^,^^5^ with p-value combination approaches I have found both HMP and Cauchy Combination Test to work rather well even at the nominal rate of 0.05; the dependencies usually have limited range (e.g. correlation matrix is banded, not full) and thus the violations of independence are not as severe. Perhaps pointing out the this aspect more prominently would make sure that the reader finds this methodology useful.

 I would like to make a number of minor suggestions that would potentially help the readability of the paper:

1. Providing an explicit example of this new class of methods would be very helpful. For example, the combination method corresponding to r=-2 marked as Levy method in Figure 6 is rather elegant and has desirable multilevel properties [personal communication with the author].
2. I find that Figure 1 and its discussion only depends on the threshold formulas not actual data. It can be moved into Section 2 to visualize the comparison of thresholds.
3. The discussion around Figure 5/excess power was not very clear to me. Perhaps a bit more detailed explanation of the notion of excess power would be helpful here.
4. Maybe include another example of data that does not have the severe dependency structure; this example does not have to be analyzed as thoroughly, but should showcase that all of these methods are practically useful.

Is the work clearly and accurately presented and does it cite the current literature?

Yes

If applicable, is the statistical analysis and its interpretation appropriate?

Yes

Are all the source data underlying the results available to ensure full reproducibility?

Yes

Is the study design appropriate and is the work technically sound?

Yes

Are the conclusions drawn adequately supported by the results?

Yes

Are sufficient details of methods and analysis provided to allow replication by others?

Yes

Reviewer Expertise:

Machine learning and data science techniques for relational structures, graphs, networks, high-dimensional and spatial datasets with an emphasis on interpretable and statistically rigorous approaches.

I confirm that I have read this submission and believe that I have an appropriate level of expertise to confirm that it is of an acceptable scientific standard.

## References

[1] : The harmonic meanp -value for combining dependent tests. *Proceedings of the National Academy of Sciences*.2019;116(4) : 10.1073/pnas.1814092116 1195-1200 10.1073/pnas.1814092116 PMC634771830610179

[2] : Cauchy combination test: a powerful test with analytic p-value calculation under arbitrary dependency structures. *J Am Stat Assoc*.2020;115(529) : 10.1080/01621459.2018.1554485 393-402 10.1080/01621459.2018.1554485 33012899PMC7531765

[3] : Kernel mean embedding based hypothesis tests for comparing spatial point patterns. *Spatial Statistics*.2020;38: 10.1016/j.spasta.2020.100459 10.1016/j.spasta.2020.100459

[4] : Combiningp -values via averaging. *Biometrika*.2020;107(4) : 10.1093/biomet/asaa027 791-808 10.1093/biomet/asaa027

[5] : Intrinsic Sliced Wasserstein Distances for Comparing Collections of Probability Distributions on Manifolds and Graphs. *Arxiv*.2020; Reference source
